# Differential efficacy of cisplatin plus pemetrexed between L858R and Del-19 in advanced *EGFR*-mutant non-squamous non-small cell lung cancer

**DOI:** 10.1186/s12885-017-3952-7

**Published:** 2018-01-02

**Authors:** Toshihiko Kaneda, Hiroshige Yoshioka, Motohiro Tamiya, Akihiro Tamiya, Akito Hata, Asukaka Okada, Takashi Niwa, Takayuki Shiroyama, Masaki Kanazu, Tadashi Ishida, Nobuyuki Katakami

**Affiliations:** 1 Department of Respiratory Medicine, Kurashiki Central Hospital, 1-1-1 Miwa, Kurashiki, Japan; 2 Department of Thoracic Malignancy, Osaka Prefectural Medical Center for Respiratory and Allergic Diseases, Habikino, Japan; 3 Department of Internal Medicine, National Hospital Organization Kinki-chuo Chest Medical Center, Sakai, Japan; 4 Division of Integrated Oncology, Institute of Biomedical Research and Innovation, Kobe, Japan; 5 Department of Respiratory Medicine, Saiseikai Suita Hospital, Suita, Japan

**Keywords:** Exon 19 deletion, L858R point mutation in exon 21, Non-squamous non-small cell lung cancer, Pemetrexed, Progression-free survival

## Abstract

**Background:**

LUX-Lung 3 showed afatinib improved progression-free survival (PFS) compared with cisplatin plus pemetrexed in patients with *epidermal growth factor receptor* (*EGFR*) mutations. In this study, chemotherapy efficacy tended to differ between patients with Leu858Arg (L858R) point mutation and Exon 19 deletion (Del-19); PFS in L858R patients (8.1 months) was greater than in Del-19 patients (5.6 months). We investigated whether there is any difference in efficacy of cisplatin plus pemetrexed between Del-19 and L858R.

**Methods:**

This study is a multicenter retrospective study. We reviewed medical records of patients who had received cisplatin plus pemetrexed as first line chemotherapy. Efficacies were evaluated between *EGFR* mutation status: Del-19 and L858R. Wild type cases were reference arm only, and not included in any statistical analysis.

**Results:**

Among 304 patients, 78 (25.7%) harbored *EGFR* mutations: Del-19 (36/78 patients, 46.2%); and L858R (42/78, 53.8%). Median PFS of L858R group (9.4 months, 95% confidence interval [CI]: 7.0–12.6) was significantly longer than Del-19 group (5.5 months, 95% CI, 3.6–8.6) (*p* = 0.049). Response rate (RR) and OS presented no significant difference between L858R and Del-19. In multivariate analysis, *EGFR* mutation status (L858R versus Del-19) was the only significant factor for longer PFS (Hazard ratio [HR]: 0.78, 95% CI: 0.62–0.98) (*p* = 0.033).

**Conclusion:**

Our study indicated better efficacy of cisplatin plus pemetrexed in L858R than in Del-19 patients. In *EGFR*-mutant NSCLC, EGFR-TKIs are undoubtedly the premier therapy. However, in second line or later settings, cisplatin plus pemetrexed regimen may confer higher efficacy for L858R patients.

## Background

Lung cancer is now one of the most common malignancies in the world. An estimated 1.8 million new lung cancer patients were diagnosed in 2012, and accounted for about 13% of total newly-diagnosed cancer patients [1]. Approximately 80% of lung cancer is histologically non-small cell lung cancer (NSCLC); most patients are already unresectable on their initial diagnosis and are selected to receive chemotherapy. Platinum doublet regimens were once the primary therapeutic choice for advanced NSCLC, but cytotoxic chemotherapies’ progress has reached a plateau. However, *epidermal growth factor receptor* (*EGFR*) tyrosine kinase inhibitors (TKIs) have improved therapeutic outcomes of *EGFR-*mutant advanced NSCLC. Exon 19 deletion (Del-19) mutation and Leu858Arg (L858R) point mutation in exon 21 are the most common *EGFR* mutations in NSCLC. Several clinical randomized phase III trials have demonstrated that *EGFR*-mutant advanced NSCLC patients treated with EGFR-TKIs obtain a longer progression-free survival (PFS) than those treated with platinum-based chemotherapy [2–7].

Combined analysis of overall survival (OS) data from two randomized phase III trials, LUX-Lung 3 and LUX-Lung 6, showed that overall survival was improved with the 2nd generation EGFR-TKI afatinib (31.7 months) over standard chemotherapy (20.7 months) for patients with Del-19 mutant NSCLC (*p* = 0.0001) [8]. These results demonstrate afatinib achieved greater effect than standard chemotherapy for Del-19 patients as a whole. Conversely, OS of patients with L858R was not significantly different between afatinib (22.1 months) and standard chemotherapy (26.9 months) (*p* = 0.16). We suppose afatinib exerts greater efficacy in Del-19, and chemotherapy exerts greater efficacy in L858R (OS of Del-19 with chemotherapy: 20.7 months, L858R with chemotherapy: 26.9 months). PFS in L858R and Del-19 patients treated with cisplatin plus gemcitabine were almost equivalent in LUX Lung 6 (5.6 months, both). However, LUX-Lung 3 results suggested cisplatin plus pemetrexed promoted longer PFS in L858R patients (8.1 months) than in Del-19 patients (5.6 months) [9]. L858R patients treated with cisplatin plus pemetrexed may obtain greater PFS than in Del-19. However, there is no verified data on this subject, and the efficacy of cisplatin plus pemetrexed for L858R is unclear now.

In this study, we retrospectively examined the efficacy of cisplatin plus pemetrexed as first line chemotherapy according to *EGFR* mutation status: Del-19 and L858R, in advanced non-squamous NSCLC.

## Methods

### Patients

We screened NSCLC patients treated with cisplatin plus pemetrexed as first line chemotherapy at participating institutions. Results of patient characteristics were analyzed using medical and radiographic records to ascertain age, gender, Eastern Cooperative Oncology Group (ECOG) performance status (PS), smoking history, clinical stage (stage IIIB, IV or recurrence), and histology. We also investigated number of induction and maintenance therapies, and post-treatment. Tumor response was retrospectively evaluated according to the Response Evaluation Criteria in Solid Tumors (RECIST) version 1.1. PFS duration was calculated from the date of initiation of cisplatin plus pemetrexed treatment to the date of disease progression or death. OS time was determined from the date of initiation of cisplatin plus pemetrexed treatment to the date of death or the last follow up on September 30th, 2015. *EGFR* mutations were analyzed using the peptide nucleic acid-locked nucleic acid PCR clamp method [10]. Since our study was a retrospective observational cohort and included no therapeutic intervention, written informed consent was waived. However, each Institutional Review Board approved this retrospective study.

### Eligibility criteria

Major inclusion criteria were ECOG PS of ≤2, NSCLC, harboring *EGFR* common mutations (Del-19, L858R) and wild type (reference arm), diagnosing histologically or cytologically non-squamous, and having received cisplatin plus pemetrexed as first line chemotherapy. We eliminated all cases having poor performance status, interstitial pneumonitis, active double cancer, and uncommon *EGFR* mutations.

### Treatment

For induction therapy, intravenous cisplatin (60–80 mg/m^2^) and intravenous pemetrexed (500 mg/m^2^) were administered every three weeks, for 4–6 cycles. During induction phase, each drug was administered until completion of 4–6 cycles, unless progressive disease (PD) or unacceptable toxicity was noted. If therapeutic efficacy was complete response (CR), partial response (PR) or stable disease (SD) at induction phase completion, then chemotherapy underwent transition to maintenance therapy. During maintenance phase, patients received intravenous pemetrexed (500 mg/m^2^) every three weeks. Maintenance therapy was administered until PD or unacceptable toxicity was noted. Chest radiography was performed every 2 to 6 weeks and chest computed tomography (CT) scans were performed every 2 to 3 cycles to evaluate treatment response and disease progression.

### Statistical analysis

Response rate (RR) and disease control rate (DCR) were compared between *EGFR* mutation positive (Del-19 and L858R) patients using Fisher’s exact test. PFS and OS curves were estimated according to the Kaplan-Meier method. PFS and OS were compared between Del-19 and L858R using log-rank test. Independent risk factors were analyzed in multivariate analysis using Cox proportional hazards model. In multivariate analysis, we selected each patients characteristics (age, gender, ECOG PS, smoking history, clinical stage, histology, and *EGFR* mutation status), and stepdown method was used in model selection to choose predictive variables. Subgroup analysis was performed between Del-19 and L858R. Wild type cases were reference arm only, and not included in any statistical analysis. *P*-values less than 0.05 were considered to be statistically significant. Statistical analysis was performed using JMP 9 software (SAS Institute Inc., Cary, NC, USA).

## Results

### Patient characteristics

Between January 2010 and December 2014, 304 patients received cisplatin plus pemetrexed as first line chemotherapy. Among 304 patients, 78 (25.7%) patients harbored *EGFR* mutations, including Del-19 (36/78 patients, 46.2%) and L858R (42/78, 53.8%). Their clinical characteristics are shown in Table 1. Median age was 64.0 years (range, 37 to 78 years). Most patients were male (216/304, 71.1%), had a good PS of 0/1 (273/304, 89.8%) and had ever smoked (219/304, 72.0%). Stage IIIB or IV (277/304, 91.1%) and adenocarcinoma (276/304, 90.8%) were predominant. However, *EGFR* mutations were predominantly female (44/78, 56.4%) and never smoker (50/78, 64.1%). L858R and Del-19 patient characteristics were not significantly different. In this investigation, histological types were limited to: adenocarcinoma; large cell carcinoma; and NSCLC-not otherwise specified (NOS). We defined these 3 histological types as non-squamous. Mea number of induction therapy cycles was 3.7 in Del-19 and 4.0 in L858R, and mean maintenance therapy cycles were 4.1 in Del-19 and 6.0 in L858R. L858R cycle numbers tended to be slightly higher than Del-19, however, no significant differences were observed. In maintenance therapy, interruption for unacceptable toxicity was undertaken in two cases, one Del-19 case and one L858R.

**Table 1:** Comparison of patient characteristics

Patient characteristics	All patients (*n* = 304)	Del-19 (*n* = 36)		L858R (*n* = 42)		*p*-value (Del-19/L858R)	Wild type (*n* = 226)	
		No. of patients	%	No. of patients	%		No. of patients	%
Age (years)								
Median (range)	64.0 (37–78)	62.5 (41–77)		66.0 (38–78)			64.0 (37–78)	
< 65	160	20	55.6	17	40.5	0.18	123	54.4
≥ 65	144	16	44.4	25	59.5		103	45.6
Gender								
Male	216	19	52.8	15	35.7	0.13	182	80.5
Female	88	17	47.2	27	64.3		44	19.5
PS (ECOG)								
0–1	273	35	97.2	40	95.2	0.65	198	87.6
2	31	1	2.8	2	4.8		28	12.4
Smoking history								
Never	85	20	55.6	30	71.4	0.15	35	15.5
Ever	219	16	44.4	12	28.6		191	84.5
Clinical stage								
IIIB, IV	277	30	83.3	35	83.3	1.00	212	93.8
Recurrence	27	6	16.7	7	16.7		14	6.2
Histology								
Adenocarcinoma	276	34	94.4	42	100	0.30	200	88.5
Large cell carcinoma	4	1	2.8	0	0		3	1.3
NSCLC-NOS	24	1	2.8	0	0		23	10.2
Induction therapy cycles								
Mean (range)	3.7(1–6)	3.7 (1–6)		4.0 (1–6)		0.23	3.6 (1–6)	
Maintenance therapy cycles								
Mean (range)	3.3(0–30)	4.1 (0–30)		6.0 (0–22)		0.17	2.7 (0–30)	

*Del-19* 19 deletion; *L858R* Leu858Arg; *PS* performance status

*ECOG* Eastern Cooperative Oncology Group

*NSCLC-NOS* non small cell lung cancer-not otherwise specified

### Treatment efficacy

Results of RR and DCR are summarized in Table 2. Overall RR was 39.7% and RRs were not significantly different between L858R and Del-19. Overall DCR was 85.9% and DCRs were not significantly different between L858R and Del-19 as well.

**Table 2:** Summary of response rate (RR) and disease control (DCR) between Del-19 and L858R

	RR (%)	*p*-value	DCR (%)	*p*-value
All patients (*n* = 78)	39.7		85.9	
EGFR mutation status				
Del-19	36.1	0.54	80.6	0.21
L858R	42.9		90.5	

*Del-19* 19 deletion; *L858R* Leu858Arg, *EGFR* epidermal growth factor receptor

**Fig. 1:**
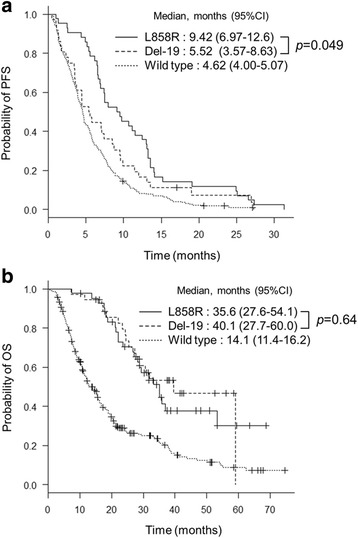
Kaplan-Meier curves for **a** progression-free survival and **b** overall survival among patients in L858R group, Del-19 group, and wild type group

**Fig. 2:**
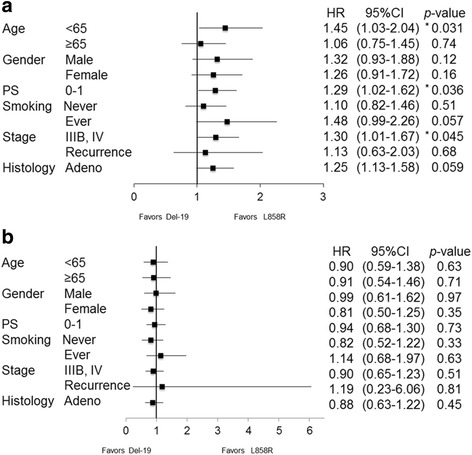
A forest plot for **a** progression-free survival hazard ratios and **b** overall survival hazard ratios comparing Del-19 group with L858R group for subgroups stratified by the indicated factors

Median PFS was significantly longer for L858R patients (9.42 months, 95% confidence interval [CI]: 6.97–12.6) than in Del-19 patients (5.52 months, 95% CI: 3.57–8.63) (*p* = 0.049) (Fig. 1a). Subgroup analyses of PFS for *EGFR* mutation status (L858R versus Del-19) are shown in Forest plot (Fig. 2a). In patients under 65 years, with good PS and clinical stage IIIB or IV, L858R mutation was favored over Del-19.

Median OS did not significantly differ between L858R (35.6 months, 95% CI: 27.6–54.1) and Del-19 (40.1 months, 95% CI: 27.7–60.0) (*p* = 0.64) (Fig. 1b). Subgroup analyses of OS for *EGFR* mutation status (L858R versus Del-19) are shown in Forest plot (Fig. 2b). No significant differences were observed.

### Multivariate analyses

Multivariate analyses were performed to identify independent risk factors using the Cox proportional hazards model. We eliminated the variables of ECOG PS and histology due to small numbers, and all wild type cases from multivariate analyses. In the results of multivariate analysis, *EGFR* mutation status remained as the only identified independent predictive factor for longer PFS (L858R: hazards ratio: 0.78, 95% CI: 0.62–0.98, *p* = 0.033) (Table 3). Multivariate analyses of OS identified clinical stage as a significant factor (IIIB, IV: hazards ratio: 2.49, 95% CI: 1.37–6.20, *p* = 0.001). However, *EGFR* mutation status was a not significant prognostic factor for OS in multivariate analysis.

**Table 3:** Multivariate analyses of progression free-survival and overall survival between Del-19and L858R (*n* = 78)

Covariate	Progression-free survival		
	Hazard ratio	95% CI	*p*-value
Age (<65 vs ≥65)	1.13	0.89–1.42	0.31
Gender (Male vs Female)	0.94	0.73–1.19	0.59
Smoking history (Never vs Ever)	0.88	0.70–1.13	0.32
Clinical stage (IIIB, IV vs Recurrence)	1.36	0.99–1.93	0.055
*EGFR* mutation status (L858R vs Del-19)	0.78	0.62–0.98	*0.033
Covariate	Overall survival		
	Hazard ratio	95% CI	*p*-value
Age (<65 vs ≥65)	1.21	0.87–1.70	0.25
Gender (Male vs Female)	1.01	0.73–1.38	0.95
Smoking history (Never vs Ever)	0.97	0.70–1.37	0.86
Clinical stage (IIIB, IV vs Recurrence)	2.49	1.37–6.20	*0.001
*EGFR* mutation status (L858R vs Del-19)	1.14	0.83–1.57	0.43

*CI* confidence interval

* p < 0.05

#### Post-treatment according to *EGFR* mutation status

Details on the post-treatment regimens are given in Table 4. There were no major differences in post-treatment between L858R and Del-19, with clear majorities of each receiving EGFR-TKIs.

**Table 4:** Post-treatment according to *EGFR* mutation status as second line between Del-19and L858R (*n* = 78)

Post-treatment	Del-19 (*n* = 36)		L858R (*n* = 42)	
	No.of patients	%	No.of patients	%
Not received	0	0%	0	0%
Received				
Platinum doublet therapy	0	0%	0	0%
Single-agent chemotherapy	1	2.8%	1	2.4%
EGFR-TKI therapy	32	88.9%	38	90.5%
Other molecular target drug therapy	2	5.5%	2	4.7%
Immunotherapy	0	0%	0	0%
Other	1	2.8%	0	0%
During first line chemotherapy	0	0%	1	2.4%

*EGFR-TKI* epidermal growth factor receptor-tyrosine kinase inhibitor

## Discussion

Pemetrexed was approved as a therapeutic drug for malignant mesotheliomas in the United States in February, 2004. For NSCLC, pemetrexed gained supplemental approval in August of that year [11]. Pemetrexed inhibits a folic acid-dependent metabolic pathway necessary for cell replication by replacing folic acid and disrupting cellular activity. Pemetrexed inhibits many enzymes: Thymidylate synthase (TS); Dihydrofolate reductase (DHFR); and Glycinate ribonucleotide formyltransferase (GARFT). Some reports indicate pemetrexed has greater efficacy in non-squamous cell carcinoma than in squamous cell carcinoma [12, 13]. This differential efficacy may be explained by the higher TS expression exhibited by squamous cell carcinoma. In squamous cell carcinoma, the higher expression of TS and activity of Skp2, the enzymes synthesizing thymidine monophosphate (TMP), decreases the efficacy of pemetrexed [14, 15].

Furthermore, in subgroup analysis of international clinical phase III trial (PROFILE1007) aimed at Anaplastic lymphoma kinase (*ALK*) positive advanced lung cancer, pemetrexed showed higher effect than docetaxel [16]. Shaw et al. reported *ALK* positive lung cancer minimally expresses TS, and pemetrexed may thus have greater efficacy [17]. Ren et al. reported low TS expression in *EGFR*-mutant NSCLC too [18]. Giovannetti et al. reported different TS gene expression level among six human NSCLC cell lines. Especially, NCI-H1650 (H1650) harboring *EGFR* mutations had lower TS gene expression than the other five NSCLC cell lines which expressed wild type *EGFR* [19]. Cells with *EGFR* mutations may have greater sensitivity to pemetrexed due to lower TS gene expression levels. However, *EGFR* mutation was not separated by Del-19 or L858R in these reports. Pemetrexed may exert greater efficacy on L858R patients if TS expression is lesser in L858R than in Del-19. Wu et al. reported pemetrexed-based chemotherapy showed a higher response and longer PFS in *EGFR*-mutant than in wild type [20]. These results may affirm our study. However, in these reports *EGFR* mutation was not separated by Del-19 or L858R as well, and no prospective study examining TS expression among differing *EGFR*-mutants has been reported.

According to preclinical data, X-ray crystallographic analysis of the domain revealed different protein conformations of Del-19 and L858R. Both vary in their activated stability by difference in conformation, and their continuation state of kinase activation after the disruption of dimerization is different also [21]. Reguart et al. reported that biological properties of Del19 and L858R mutations differ, with different patterns of *EGFR* amplification and *EGFR* autophosphorylation between cell lines containing each mutation [22]. Other experimental reports show that biomedically, L858R and Del-19 may be two different things [23, 24]. Carey et al. compared the proliferation abilities of induced wild type, Del-19, and L858R NR6 fibroblasts [25]. According to this report, there are differences in cell proliferation ability among wild type, Del-19 and L858R. Especially in Del-19, high cell proliferation ability has been confirmed. Therefore, Del-19 may be faster in progression speed than L858R. Accordingly, L858R and Del-19 may respond differently to pemetrexed.

Various resistance mechanisms to EGFR-TKI treatments, such as *ErBB* family receptor amplification or other RTK co-amplification may be involved in the differential efficacy between L858R and Del-19. Yu et al. reported that frequencies of *Met*-amplification and *HER*-amplification did not differ between L858R and Del-19, although sample size was small [26]. The possible differences between Del19 and L858R require further research.

In comparison with the results of LUX-Lung 3, PFS of our study was similar. In Del-19, PFS of LUX-Lung 3 was 5.6 months versus 5.52 months in our study. In L858R, PFS of LUX-Lung 3 was 8.1 months versus 9.42 months in our study. The results of our study showed reproducibility. Our study demonstrated significant difference in PFS between L858R and Del-19 treated with pemetrexed, but did not reveal significant difference in OS. EGFR-TKIs were administered post-pemetrexed in approximately 90% of each mutation type, and most have reported higher EGFR-TKI efficacy in Del-19 than in L858R [4–6, 8]. This “catch-up” effect may explain similar OS between L858R and Del-19. Our study covered wild type as reference group in this study. Wild type PFS was 4.62 months, in accordance with past reports [27]. In multivariate analysis of OS, a significant difference was found between recurrence and advaced (clinical stage IIIB or IV) NSCLC. Yoshioka et al**.** reported that recurrent lung cancer had good prognosis and our study agrees in this respect [28].

Our study presents a few limitations. First, it is retrospective. Second, our sample size is relatively small. However, we were able to examine and obtain pure data by only including first line cisplatin plus pemetrexed treated patients and excluding other regimens (for instance, carboplatin-based or addition of bevacizumab), mirroring the strict patient selection of LUX-Lung 3. Finally, we were not able to perform TS expression immunostaining to conclude any causal link between *EGFR* mutation type, TS expression and treatment efficacy in this study. Further studies are warranted.

This study is the first report showing a significantly greater efficacy of cisplatin plus pemetrexed in L858R than in Del-19 between *EGFR-*mutant NSCLC chemotherapy naïve patients. In this study, we examined cisplatin plus pemetrexed regimen in first line chemotherapy, similar to LUX-lung studies. In *EGFR*-mutant NSCLC, EGFR-TKIs are undoubtedly the premier therapy, with limitations. Third generation EGFR-TKI after acquired resistance to first or second generation EGFR-TKIs represents an efficacious treatment [29], but only in the roughly 50% of patients who express T790 M mutation [30]. Immunotherapy has also attracted investigation in NSCLC [31–33], but it has shown reduced efficacy against *EGFR*-mutants [34, 35]. Thus, cytotoxic chemotherapy still has a role in treating patients who cannot benefit from further EGFR-TKI exposure or immunotherapy. Furthermore, administering platinum doublet chemotherapy alternating with EGFR-TKI produced longer survivals among *EGFR*-mutants than EGFR-TKI alone [28]. In addition, some report that *EGFR*-mutants may be more likely to benefit from cytotoxic chemotherapy than wild type [36, 37].

## Conclusion

Our study indicated better efficacy of cisplatin plus pemetrexed in L858R than in Del-19 patients. In *EGFR*-mutant NSCLC, EGFR-TKIs are undoubtedly the premier therapy. However, in second line or later settings, cisplatin plus pemetrexed regimen may confer higher efficacy for L858R patients.

## Abbreviations

ALK: Anaplastic lymphoma kinase
CI: confidence interval
CR: complete response
CT: computed tomography
DCR: disease control rate
Del-19: 19 deletion
DHFR: Dihydrofolate reductase
ECOG: Eastern Cooperative Oncology Group
EGFR: Epidermal growth factor receptor
GARFT: Glycinate ribonucleotide formyltransferase
L858R: Leu858Arg
NOS: not otherwise specified
NSCLC: non-small cell lung cancer
OS: overall survival
PD: progressive disease
PFS: progression-free survival
PR: partial response
PS: performance status
RECIST: Response Evaluation Criteria in Solid Tumors
RR: Response rate
SD: stable disease
TKIs: tyrosine kinase inhibitors
TS: Thymidylate synthase

## References

[1] Torre LA, Bray F, Siegel RL, Ferlay J, Lortet-Tieulent J, Jemal A (2015). Global cancer statistics, 2012. CA Cancer J Clin.

[2] Mitsudomi T, Morita S, Yatabe Y, Negoro S, Okamoto I, Tsurutani J (2010). Gefitinib versus cisplatin plus docetaxel in patients with non-small-cell lung cancer harbouring mutations of the epidermal growth factor receptor (WJTOG3405): an open label, randomised phase 3 trial. Lancet Oncol.

[3] Maemondo M, Inoue A, Kobayashi K, Sugawara S, Oizumi S, Isobe H (2010). Gefitinib or chemotherapy for non-small-cell lung cancer with mutated EGFR. N Engl J Med.

[4] Zhou C, YL W, Chen G, Feng J, Liu XQ, Wang C (2011). Erlotinib versus chemotherapy as first-line treatment for patients with advanced EGFR mutation-positive non-small-cell lung cancer (OPTIMAL, CTONG-0802): a multicentre, open-label, randomised, phase 3 study. Lancet Oncol..

[5] Rosell R, Carcereny E, Gervais R, Vergnenegre A, Massuti B, Felip E (2012). Erlotinib versus standard chemotherapy as first-line treatment for European patients with advanced EGFR mutation-positive non-small-cell lung cancer (EURTAC): a multicentre, open-label, randomised phase 3 trial. Lancet Oncol..

[6] Sequist LV, Yang JC, Yamamoto N, O'Byrne K, Hirsh V, Mok T (2013). Phase III study of afatinib or cisplatin plus pemetrexed in patients with metastatic lung adenocarcinoma with EGFR mutations. J Clin Oncol.

[7] Wu YL, Zhou C, Hu CP, Feng J, Lu S, Huang Y (2014). Afatinib versus cisplatin plus gemcitabine for first-line treatment of Asian patients with advanced non-small-cell lung cancer harbouring EGFR mutations (LUX-lung 6): an open-label, randomised phase 3 trial. Lancet Oncol..

[8] Yang JC, Wu YL, Schuler M, Sebastian M, Popat S, Yamamoto N (2015). Afatinib versus cisplatin-based chemotherapy for EGFR mutation-positive lung adenocarcinoma (LUX-lung 3 and LUX-lung 6): analysis of overall survival data from two randomized, phase 3 trials. Lancet Oncol..

[9] European Medicines Agency. Giotrif (afatinib) film-coated tablets: EU summary of product characteristics. 2013. http://www.ema.europa.eu/docs/en_GB/document_library/EPAR_-_Product_Information/human/002280/WC500152392.pdf

[10] Nagai Y, Miyazawa H, Huqun TT, Udagawa K, Kato M (2005). Genetic heterogeneity of the epidermal growth factor receptor in non-small cell lung cancer cell lines revealed by a rapid and sensitive detection system, the peptide nucleic acid-locked nucleic acid PCR clamp. Cancer Res.

[11] FDA approval for pemetrexed disodium –National Cancer Institute- http://www.cancer.gov/about-cancer/treatment/drugs/fda-pemetrexed-disodium.

[12] Scagliotti GV, Parikh P, von Pawel J, Biesma B, Vansteenkiste J, Manegold C (2008). Phase III study comparing cisplatin plus gemcitabine with cisplatin plus pemetrexed in chemotherapy-naive patients with advanced-stage non-small-cell lung cancer. J Clin Oncol.

[13] Kubota K, Niho S, Enatsu S, Nambu Y, Nishiwaki Y, Saijo N (2009). Efficacy differences of pemetrexed by histology in pretreated patients with stage IIIB/IV non-small cell lung cancer: review of results from an open-label randomized phase II study. J Thorac Oncol.

[14] Nicolson MC, Fennell DA, Ferry D, O'Byrne K, Shah R, Potter V (2013). Thymidylate synthase expression and outcome of patients receiving pemetrexed for advanced nonsquamous non-small-cell lung cancer in a prospective blinded assessment phase II clinical trial. J Thorac Oncol.

[15] Ceppi P, Volante M, Saviozzi S, Rapa I, Novello S, Cambieri A (2006). Squamous cell carcinoma of the lung compared with other histotypes shows higher messenger RNA and protein levels for thymidylate synthase. Cancer.

[16] Shaw AT, Kim DW, Nakagawa K, Seto T, Crinó L, Ahn MJ (2013). Crizotinib versus chemotherapy in advanced ALK-positive lung cancer. N Engl J Med.

[17] Shaw AT, Varghese AM, Solomon BJ, Costa DB, Novello S, Mino-Kenudson M (2013). Pemetrexed-based chemotherapy in patients with advanced, ALK-positive non-small cell lung cancer. Ann Oncol.

[18] Ren S, Chen X, Kuang P, Zheng L, Su C, Li J (2012). Association of EGFR mutation or ALK rearrangement with expression of DNA repair and synthesis genes in never-smoker women with pulmonary adenocarcinoma. Cancer.

[19] Giovannetti E, Lemos C, Tekle C, Smid K, Nannizzi S, Rodriguez JA (2008). Molecular mechanisms underlying the synergistic interaction of erlotinib, an epidermal growth factor receptor tyrosine kinase inhibitor, with the multitargeted antifolate pemetrexed in non-small-cell lung cancer cells. Mol Pharmacol.

[20] SG W, Yang CH, CJ Y, Lee JH, Hsu YC, Chang YL (2011). Good response to pemetrexed in patients of lung adenocarcinoma with epidermal growth factor receptor (EGFR) mutations. Lung Cancer.

[21] Cho J, Chen L, Sangji N, Okabe T, Yonesaka K, Francis JM (2013). Cetuximab response of lung cancer-derived EGF receptor mutants is associated with asymmetric dimerization. Cancer Res.

[22] Reguart N, Remon J (2015). Common EGFR-mutated subgroups (Del19 ⁄ L858R) in advanced non-small-cell lung cancer: chasing better outcomes with tyrosinekinase inhibitors. Future Oncol.

[23] Zhang X, Pickin KA, Bose R, Jura N, Cole PA, Kuriyan J (2007). Inhibition of the EGF receptor by binding of MIG6 to an activating kinase domain interface. Nature.

[24] Zhu JQ, Zhong WZ, Zhang GC, Li R, Zhang XC, Guo AL (2008). Better survival with EGFR exon 19 than exon 21 mutations in gefitinib-treated non-small cell lung cancer patients is due to differential inhibition of downstream signals. Cancer Lett.

[25] Carey KD, Garton AJ, Romero MS, Kahler J, Thomson S, Ross S (2006). Kinetic analysis of epidermal growth factor receptor somatic mutant proteins shows increased sensitivity to the epidermal growth factor receptor tyrosine kinase inhibitor, erlotinib. Cancer Res.

[26] Yu HA, Arcila ME, Rekhtman N, Sima CS, Zakowski MF, Pao W (2013). Analysis of tumor specimens at the time of acquired resistance to EGFR-TKI therapy in 155 patients with EGFR-mutant lung cancers. Clin Cancer Res.

[27] Kang EJ, Min KH, Hur GY, Lee SY, Shim JJ, Kang KH (2015). Comparison of the efficacy between Pemetrerxed plus platinum and non-Pemetrexed plus platinum as first-line treatment in patients with wild-type epidermal growth factor receptor nonsquamous non-small cell lung cancer: a retrospective analysis. Chemotherapy.

[28] Yoshioka H, Mitsudomi T, Morita S, Yatabe Y, Negoro S, Okamoto I, et al. Final overall survival results of WJTOG 3405, a randomized phase 3 trial comparing gefitinib (G) with cisplatin plus docetaxel (CD) as the first-line treatment for patients with non-small cell lung cancer (NSCLC) harboring mutations of the epidermal growth factor receptor (EGFR). J Clin Oncol. 2014;32:5s (suppl; abstr 8117).

[29] Mok TS, Y-L W, Ahn M-J, Garassino MC, Kim HR, Ramalingam SS (2016). Osimertinib or platinum-Pemetrexed in EGFR T790M-positive lung cancer. N Engl J Med.

[30] Nosaki K, Satouchi M, Kurata T, Yoshida T, Okamoto I, Katakami N (2016). Re-biopsy status among non-small cell lung cancer patients in Japan: a retrospective study. Lung Cancer.

[31] Brahmer J, Reckamp KL, Baas P, Crinò L, Eberhardt WE, Poddubskaya E (2015). Nivolumab versus docetaxel in advanced squamous-cell non-small-cell lung cancer. N Engl J Med.

[32] Borghaei H, Paz-Ares L, Horn L, Spigel DR, Steins M, Ready NE (2015). Nivolumab versus docetaxel in advanced nonsquamous non-small-cell lung cancer. N Engl J Med.

[33] Reck M, Rodríguez-Abreu D, Robinson AG, Hui R, Csőszi T, Fülöp A (2016). Pembrolizumab versus chemotherapy for PD-L1-positive non-small-cell lung cancer. N Engl J Med.

[34] Gainor JF, Shaw AT, Sequist LV, Fu X, Azzoli CG, Piotrowska Z (2016). EGFR mutations and ALK rearrangements are associated with low response rates to PD-1 pathway blockade in non-small cell lung cancer: a retrospective analysis. Clin Cancer Res.

[35] Spigel DR, Schrock AB, Fabrizio D, Frampton GM, Sun J, He J, et al. Total mutation burden (TMB) in lung cancer (LC) and relationship with response to PD-1/PD-L1 targeted therapies. J Clin Oncol. 2016;34 (suppl; abstr 9017).

[36] YL W, Chu DT, Han B, Liu X, Zhang L, Zhou C (2012). Phase III, randomized, open-label, first-line study in Asia of gefitinib versus carboplatin/paclitaxel in clinically selected patients with advanced non-small-cell lung cancer: evaluation of patients recruited from mainland China. Asia Pac J Clin Oncol.

[37] Zhou C, YL W, Chen G, Liu X, Zhu Y, Lu S (2015). BEYOND: a randomized, double-blind, placebo-controlled, multicenter, phase III study of first-line carboplatin/paclitaxel plus bevacizumab or placebo in Chinese patients with advanced or recurrent nonsquamous non-small-cell lung cancer. J Clin Oncol.

